# Uncertainty monitoring in Eurasian jays (*Garrulus glandarius*)

**DOI:** 10.1007/s10071-025-01960-3

**Published:** 2025-05-15

**Authors:** M. Loconsole, A. K. Schnell, E. Garcia-Pelegrin, N. S. Clayton

**Affiliations:** 1 Department of General Psychology, University of Padua, Padua, Italy; 2 Department of Psychology, University of Cambridge, Cambridge, UK; 3 Department of Psychology, National University of Singapore, Singapore, Singapore

**Keywords:** Avian cognition, Eurasian jays, Individual differences, Metacognition, Self-awareness, Uncertainty monitoring

## Abstract

Metacognition– namely the capacity to reflect on one’s own cognitive processes - provides animals with numerous evolutionary advantages. Metacognition abilities encompass enhanced decision-making in uncertain situations, more efficient resource management, error detection and correction, and improved problem-solving skills. Here, we investigate how Eurasian jays, *Garrulus glandarius*, monitor uncertainty through a working memory food-retrieval task. In this task, a desirable food item is hidden under one of two cups, which are then shuffled either once (easy treatment) or several times (difficult treatment). The jays then choose to either engage in locating the food or opt out by selecting a third cup that offers a less preferred food reward. Our findings reveal that the difficulty of the task significantly influenced the jays’ choice, with a higher tendency to opt out during difficult trials. Individual performance analysis revealed that when jays that typically opted out of difficult trials chose to engage instead, they exhibited significant accuracy. This suggests their decisions were guided by a confidence assessment of their knowledge. Overall, our study indicates that Eurasian jays possess metacognitive abilities that enable them to evaluate their own certainty and make strategic decisions based on perceived task difficulty and confidence in their knowledge. These capabilities likely confer advantages in natural settings, such as caching behaviours, allowing jays to make well-informed decisions about when to store or retrieve food based on environmental cues and internal assessments of uncertainty.

**Supplementary Information:**

The online version contains supplementary material available at 10.1007/s10071-025-01960-3.

The monitoring of one’s own mental state is a fundamental feature of metacognition (Nelson 1992). Possessing a ‘feeling-of-knowing’ allows an individual to implement adaptive behaviours, either by engaging in a task or by deferring a response and seeking more information. Traditional measures of metacognition and uncertainty monitoring in human studies mainly consist of verbal self-reports, which are not suitable for comparative studies. Instead, comparative studies rely on a series of behavioural, non-verbal tests. The classical paradigm consists of two separate components: (i) a task that requires some cognitive effort (e.g., perceptual discrimination, memory test) where a reward is delivered only after providing the correct response; and (ii) an opt out response that always leads to a reward, albeit less valuable than the one obtained by engaging in the task. By manipulating the difficulty of the main task, it is possible to induce a state of uncertainty in the subject. If animals possess a conscious ‘feeling-of-knowing’, we could expect them to selectively decline the error-risking trials.

The capability to selectively opt out of an experimental task when it is too difficult to solve has been widely reported in mammals (e.g., primates (Suda-King 2008; Suda-King et al. 2013), monkeys (Beran et al. 2014; Hampton 2001; Washburn et al. 2006), rats (Foote and Crystal 2007; Yuki and Okanoya 2017), and dolphins (Smith et al. 1995). Although studies have investigated uncertainty monitoring in non-mammalian species, their results are ambiguous. Data on pigeons failed to report uncertainty monitoring (Roberts et al. 2009), or to exclude simpler explanation for the subjects’ performance, concluding that the birds’ behaviour was not functionally comparable to that of mammals (Inman and Shettleworth 1999; Sole et al. 2003). However, promising evidence was reported in two different studies: one testing pigeons and bantams (Nakamura et al. 2011), and one testing large-billed crows (Goto and Watanabe 2012). In these studies, birds demonstrated uncertainty monitoring in retrospective judgment tasks but failed in prospective memory judgement, which is more closely related to self-awareness and metacognitive abilities, as it requires a judgement on their own knowledge before answering the test. Although not yet conclusive, these studies suggest avian species possess self-reflective cognitive abilities, warranting further investigation. Previous work on metacognition in birds suggests that jays could be a promising candidate for this goal (Nakamura et al. 2011). In fact, these birds have been previously tested for several cognitive capabilities that strictly relate to self-awareness. They can solve memory tasks requiring the retrieval of a hidden reward and remember and create expectations about the attended food (Clayton and Dickinson 1999; Schnell et al. 2021). They are known to represent the mental states of other individuals, and they adjust their behaviour to account for the presence of others, either to avoid pilfering after caching (Dally et al. 2006; Emery and Clayton 2001; Grodzinski et al. 2012; Legg and Clayton 2014; Shaw and Clayton 2012, 2013), or to better accommodate the other’s needs during food sharing (Ostojić et al. 2013). They have shown the capability of forming perspective judgements about future events (Cheke and Clayton 2012; Clayton and Dickinson 1998, 1999; Raby et al. 2007). Lastly, they react to uncertainty and actively seek more information when it is insufficient (Watanabe et al. 2014; Watanabe and Clayton 2016).

Here, we test whether Eurasian jays, *Garrulus glandarius*, can provide uncertainty responses and adjust their behaviour depending on their evaluation of their own knowledge in a food-retrieving working memory task. Birds were presented with two identical upside-down cups and witnessed the experimenter placing a highly palatable food reward in one of the two cups. The experimenter then flipped the cups and shuffled them, either following a one-step shuffle (i.e., the cups were moved from their original place only once), or a multiple-steps shuffle (i.e., the cups were moved from their original place three to four times). Birds could lift one of the two cups, and retrieve the reward hidden underneath or opt out from the task by lifting a different cup placed in one corner of the apparatus, previously associated with a less palatable food item.

Direct evidence from an avian species would help broaden the phylogenetic framework of metacognitive capacities related to self-awareness, illuminating important issues in the study of cognition.

## Materials and methods

### Ethical note

The subjects were 5-year-old Eurasian jays, *Garrulus glandarius*, from a long-term, stable social group of 16 birds, housed in an outdoor aviary (20 × 30 × 4 m) at the Sub-Department of Animal Behaviour, University of Cambridge, UK. Birds were provided with a maintenance diet consisting of a mixture of soaked dog food, vegetables, eggs, seeds, and fruits, and had *ad libitum* access to water. During the experiment they received extra food (i.e., cheese, waxworms, or peanuts) as a reward.

The maintenance diet was removed from the aviary one hour prior to testing, whereas water always remained available.

Subjects could start the daily training or testing session on a voluntary basis by entering an open flap door (0.5 × 0.5 m) that connected the outdoor aviary with the indoor rooms (2 × 1 × 2 m indoor compartments), which were linked to the final compartment, serving as the testing room. Voluntary participation ensured that animals were sufficiently motivated to engage with the task. Subjects participated in training and testing individually, which allowed us to control for direct influences from other birds, such as hierarchy in accessing food or food sharing behaviours. During the experimental procedure, the bird was required to sit on a perch in front of a testing window. The experimenter stood in an adjacent compartment that was connected to the testing room via a small window, so that the bird could interact with the experimenter without seeing their face.

All the experimental procedures were approved by the University of Cambridge and conducted under a non-regulated procedure (no. zoo72/19).

### Subjects and housing conditions

We collected data from 9 subjects (4 males: Godot, Homer, Poe, and Roland; and 5 females: Chinook, Dolcinea, Jaylo, Penny, and Stuka) over a period of four months (Nov– Feb). Two subjects (Roland and Penny) lost interest in the experiment and were subsequently excluded from the study due to insufficient data collection. Hence, our final sample consisted of 7 subjects.

### Pre-training on object performance

All subjects had participated in previous experiments that demonstrated they could successfully solve object permanence tasks (Garcia-Pelegrin et al. 2021; Schnell et al. 2021). Before starting the experimental procedure, jays underwent a brief refresh phase to assess their current ability to retrieve hidden objects. While the bird sat on the perch, the experimenter presented a worm, inserted the worm in an opaque cup, and then flipped the cup on the table. A string was attached to the base of the cup, allowing the bird to lift the cup by holding the string in its beak. All subjects successfully retrieved the food from under the cup in 10 out of 10 consecutive trials.

### Food preference assessment

Prior to testing, we assessed individual food preferences between three commonly consumed foods: waxworms, peanuts, and cubes of cheese (0.5 × 0.5 cm). These data were also employed in another study conducted during the same period (Schnell et al. 2021). Each subject completed three sessions of 12 trials each. A time window of a minimum of 24 h, to a maximum of 38 h passed between sessions. At the beginning of each trial the experimenter presented a combination of the different food items (i.e., waxworm vs. peanut, waxworm vs. cheese, cheese vs. peanut) on a raised platform placed in front of the perch. The two food items were simultaneously placed on the platform, approximately 15 cm apart and equidistant from the bird’s position. The spatial position (left/right) of each item was counterbalanced between trials. Once the bird chose one item, the other one was removed by the experimenter. The bird was then given approximately 30 s to consume the selected food, or to hide it in its throat pouch, then a new trial was started. This procedure allowed us to identify the most preferred and the second preferred food for each individual bird (Table 1).

**Table 1:** Food preference for each subject. We assessed food preferences for each subject and selected the most preferred food and the second preferred food out of three commonly used food rewards (i.e., peanuts, waxworms, and cheese)

Subject	Most preferred food	Second preferred food
Chinook	Waxworm	Peanut
Dolcinea	Waxworm	Cheese
Godot	Waxworm	Cheese
Homer	Peanut	Waxworm
Jaylo	Waxworm	Cheese
Poe	Waxworm	Cheese
Stuka	Peanut	Waxworm

### Training

**Fig. 1:**
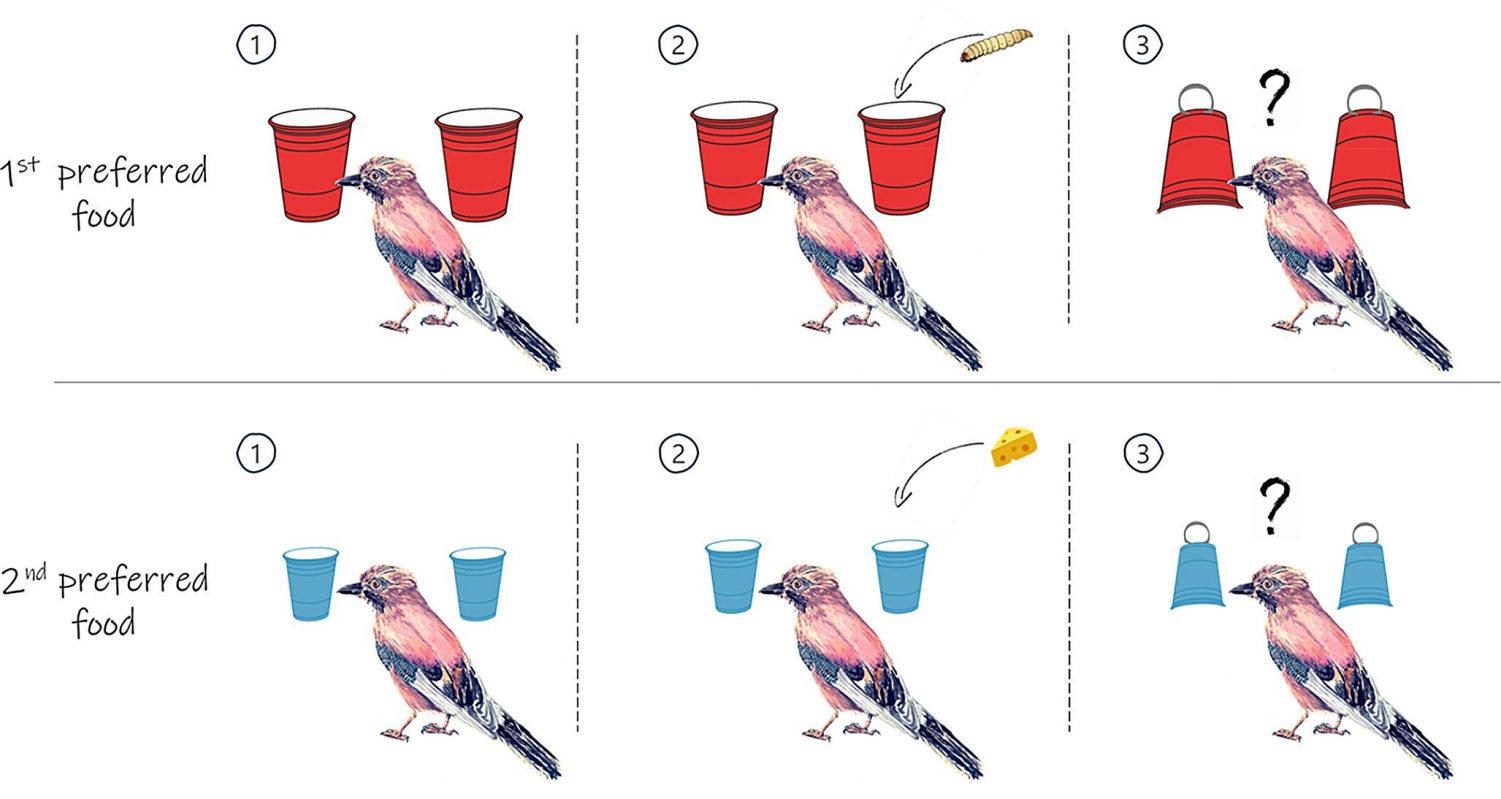
Training procedure

Each subject was trained to associate a cup (i.e., either a smaller blue or a larger red cup) with a specific food reward (i.e., the second preferred or the most preferred food, respectively, Fig. 1). We used the first and second preferred food items, rather than the most and least preferred, to maintain motivation and ensure that the blue cup remained a viable option. This approach allowed birds to weigh certainty against reward quality without one option being consistently avoided due to low value.While the bird sat on the perch, the experimenter put two identical up-turned cups in front of the subject, 15 cm apart and equidistant from the bird. The bird observed the experimenter bait one of the two cups with a food reward. The experimenter then simultaneously flipped both cups upside-down and allowed the bird to lift one of them to retrieve the hidden food. In half of the trials, both cups were smaller in size and blue in colour. Blue cups were always baited with the second preferred food. In the other half of the trials, both cups were larger in size and red in colour. Red cups were always baited with the most preferred food. After the bird retrieved and consumed the food reward, a new trial began. If the bird lifted the wrong cup (i.e., the one without a food reward), it received no food, and a new trial was started. Birds could participate in a maximum of 12 trials per day, in which red- and blue- cups trials were pseudorandomly alternated. Upon completing 48 successful trials, training was considered complete, and birds could proceed to the testing phase.

### Test

A minimum of 24 h elapsed between training and test phases. At test, as for the other procedures, birds were free to enter the experimental room at will and equally free to leave at any point. No bird ever skipped an entire day of testing or training — all birds entered the room daily throughout the data collection period. Birds typically performed a variable number of trials per session, and they were free to leave the setup at any time. This natural regulation ensured that each session remained within the bird’s own tolerance and motivation levels, effectively preventing overexposure or disengagement and maintaining reliable task performance.

**Fig. 2:**
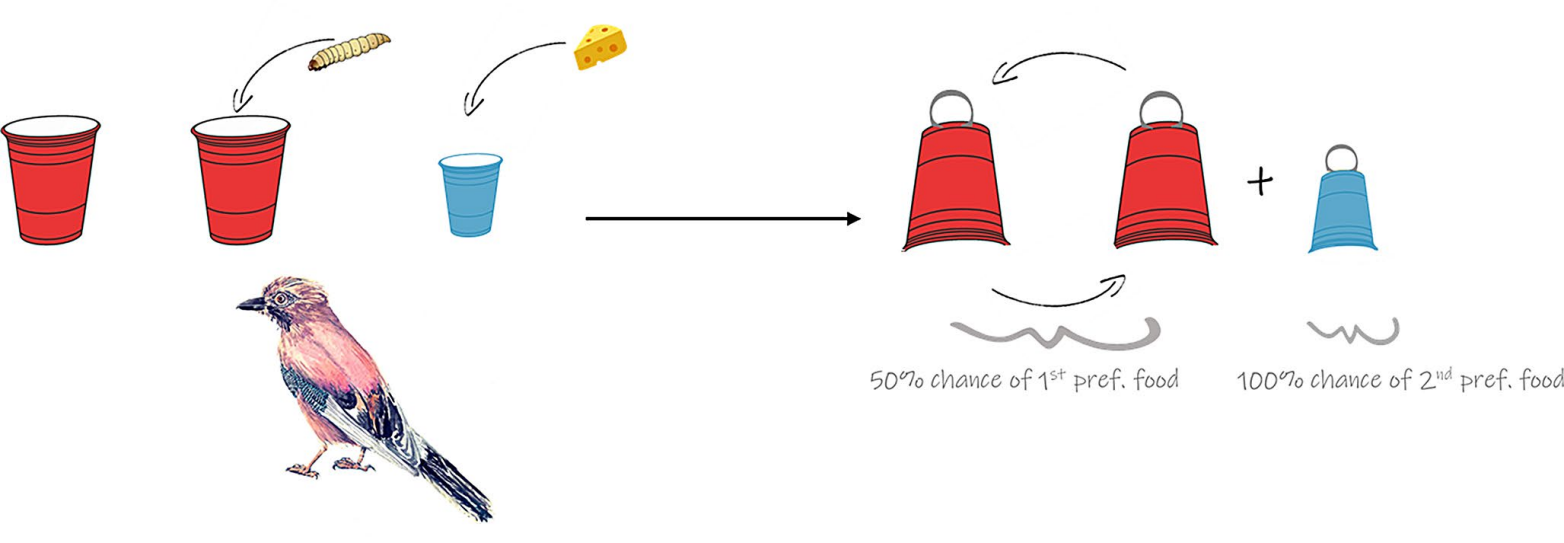
Test procedure

During the test, the bird was presented with two identical red cups placed on a table in front of the perch. Additionally, a single blue cup was placed on one side of the table on the same level as the two red cups in their final displacement. The experimenter visibly placed the second preferred reward inside the blue cup and flipped it upside-down. The spatial position of the blue cup (left or right side) was counterbalanced between trials. Next, the experimenter placed the most preferred reward in one of the two red cups, flipped both cups upside-down, and shuffled them (Fig. 2). Birds encountered either a one-step shuffle (i.e., the cups were moved from their original position only once), or a multiple-step shuffle (i.e., the cups were moved from their original position three to four times). The direction of the shuffle (clockwise or counterclockwise), and the spatial position of the baited cup were counterbalanced between trials. After shuffling the cups, the experimenter allowed the bird to lift only one cup (either the blue one or one of the red ones). If the bird chose the correct red cup or the blue cup, it was allowed to consume the reward underneath, and a new trial was immediately started after a brief pause for the experimenter to prepare (approximately 30 s). If the bird chose the incorrect red cup, all cups were promptly removed without giving the bird the opportunity to lift another, and, as with correct trials, a new trial was immediately initiated.

The test phase lasted between 10 and 14 days for each subject. A daily session never exceeded 12 trials, comprising 6 single-step and 6 multiple-step shuffle conditions, which were alternated pseudo-randomly. In total, each subject completed 88 trials (i.e., 44 for each condition). Testing sessions were recorded using a GoPro^®^ Hero 6 video-camera and analysed offline.

### Data scoring and statistical analyses

All trials were analysed using the video recordings. We coded: (i) whether each individual engaged in the task or opted out (choice of the blue cup); (ii) whether after engaging in the task birds selected the correct (the one containing the food reward), or the wrong cup. To assess inter-rater reliability, another experimenter independently scored a randomly selected sample comprising 20% of the trials, ensuring a balanced representation of both treatments (easy vs. difficult) and birds. The reliability was excellent for choice (engage vs. opt out) (Cohen’s Kappa = 0.95), as well as for accuracy of their choices during engagement (Cohen’s Kappa = 0.96).

Statistical analyses were completed using RStudio for Mac (version 2024.04.0 + 735). We analysed the data by employing Generalized Linear Mixed Models (GLMM) to examine the effect of treatment (easy vs. difficult) on the engagement decisions of birds, as well at the accuracy when engaged (mixed_model function, GLMMadaptive package). Treatment (easy vs. difficult) was included as a fixed effect, while subject ID was included as a random intercept to account for individual variation and repeated measures. Session number or time was not included because trials were pseudorandomized and balanced across sessions, ensuring that potential session effects were not systematically confounded with treatment. To check our model’s assumptions, we used the DHARMa package (Hartig and Lohse 2022). Our model successfully converged and displayed a 98% confidence interval. The assumption checks indicated no deviation from the expected distribution, nor quantile deviations of the residuals against the predicted values. Subsequently, individual bird performance in the difficult treatment was analysed by assessing the proportion of correct responses using a one-tailed t-test to compare responses against chance levels. Additionally, opt out responses for each bird were similarly evaluated.

Subsequently, individual bird performance across both the easy and difficult treatments was analyzed by assessing the proportion of correct responses using a one-tailed binomial test to compare responses against chance levels. This approach was chosen because the hypotheses were based on predictions that birds would perform significantly better than chance in both easy and difficult trials (i.e., greater than 50% correct responses). Similarly, opt-out responses were evaluated using the binomial test. A one-tailed test was appropriate because we specifically predicted that the birds would exhibit higher rates of correct responses and opt-outs than would be expected by chance, reflecting their task performance and engagement under varying levels of difficulty.

For all the analyses we set the alpha level to 0.05.

To assess individual metacognitive decisions regarding task engagement versus opting out, a binomial test was conducted for each subject. The number of opt-out trials (coded as 1) and incorrect trials (coded as 0) were compared. The null hypothesis for the binomial test was that the proportion of opt-out trials was equal to the proportion of incorrect trials (*p* =.5). This analysis aimed to determine whether subjects were more likely to opt out or engage with the task, even when they might be unsure of their ability to answer correctly, reflecting their metacognitive evaluation of task difficulty. The test was performed separately for each subject and treatment condition (easy versus difficult), with significance assessed using a threshold of *p* <.05.

## Results

### Food preferences

We created three separate contingency tables—one for each food pairing—and analyzed food preferences using chi-square tests. The tables are included as supplementary material. All birds showed a strong preference (over 90%) for their top-choice food, except for one individual (Poe). Poe exhibited a noticeable preference for waxworms over cheese, choosing the former in approximately 67% of trials; however, this preference was not statistically significant in the chi-square analysis.

### Correct versus incorrect

**Fig. 3:**
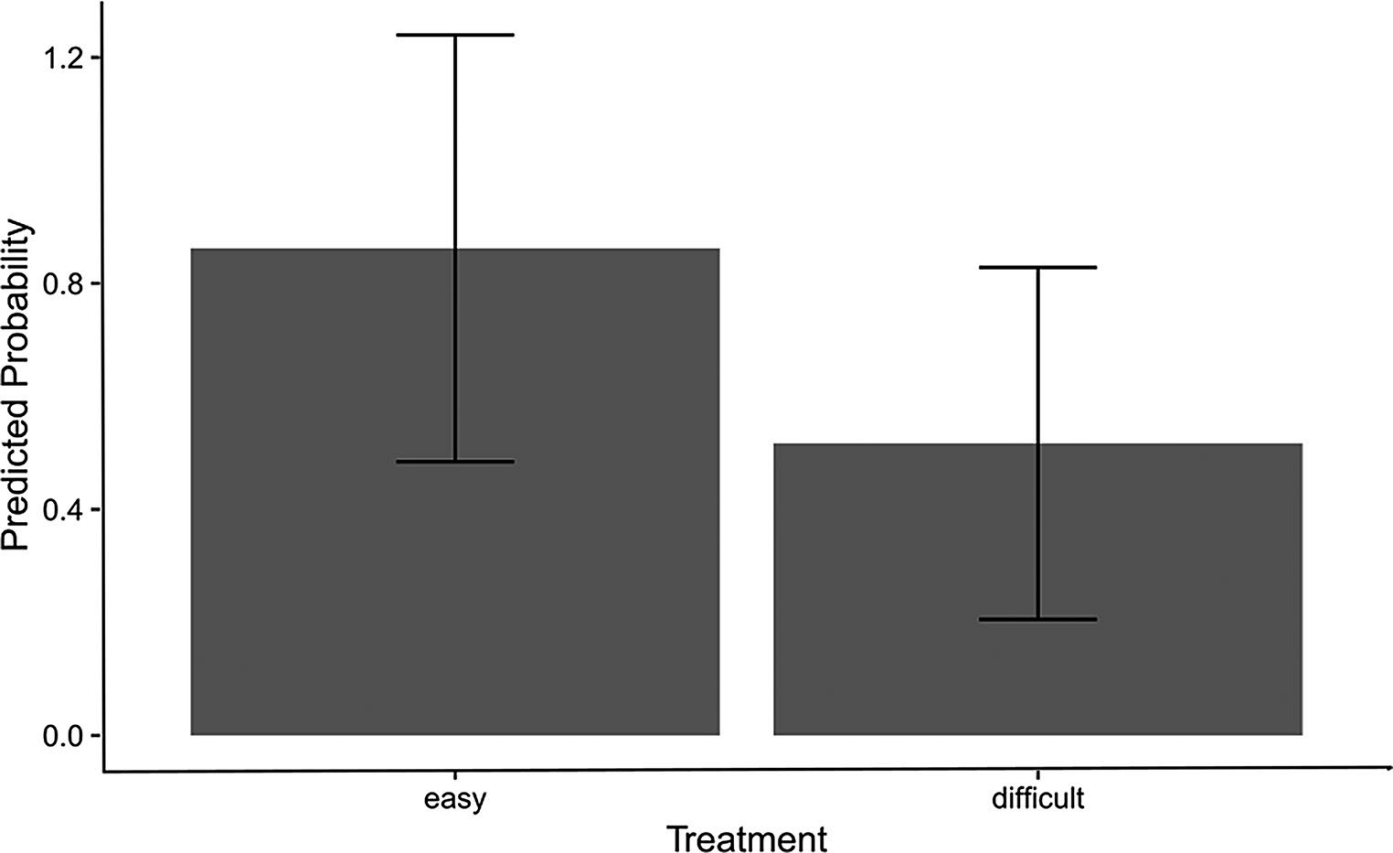
Predicted probability of correct choices by treatment difficulty: This figure shows the predicted probability of making correct choices when engaged in the tasks (easy vs. difficult). Error bars (whiskers) showing the standard error of the mean

The analysis revealed that treatment (easy *versus* difficult) had a significant positive effect on the outcome. The estimated effect size (coefficient) was 1.77, indicating that an increase of one unit in treatment is associated with an increase in the log odds of the outcome by approximately 1.77. The standard error of this estimate was 0.22, demonstrating a high degree of precision in the estimation. The z-value of 8.24 suggests that the effect is highly statistically significant (*p* <.001). These results strongly suggest that the treatment is an important predictor of the whether the outcome was correct or incorrect, and indicates that the difficult treatment is in fact of higher complexity than the easy one (Fig. 3).

### Engage versus opt-out

**Fig. 4:**
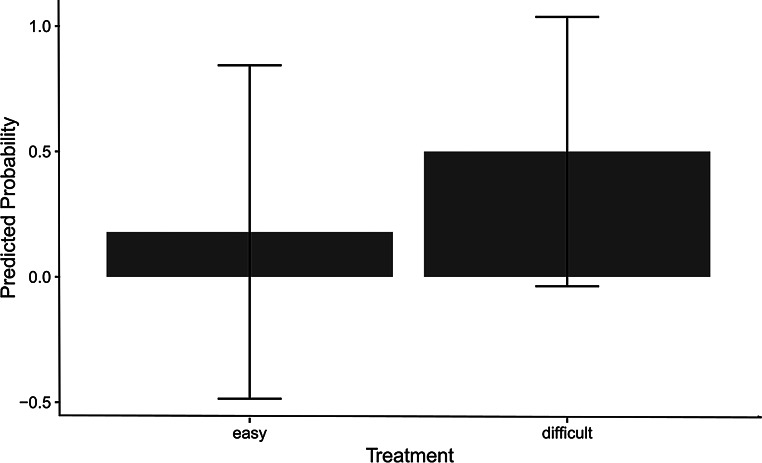
Predicted probability of choosing to opt out across treatments: This figure shows the predicted probability of opting out as a function of treatment difficulty (easy vs. difficult). Bars on the plot display the probabilities, with error bars (whiskers) showing the standard error of the mean

The analysis revealed that treatment (easy *versus* difficult) had a significant positive effect on the response. The estimated effect size (coefficient) was 1.72, indicating that an increase of one unit in treatment is associated with an increase in the log odds of the outcome by approximately 1.72. The standard error of this estimate was 0.186, demonstrating a high degree of precision in the estimation. The z-value of 9.21 suggests that the effect is highly statistically significant (*p* <.001). These results strongly suggest that the treatment is an important predictor of the response between engage or opt-out and shows that birds are more likely to opt out in the difficult treatment than in the easy one (Fig. 4).

### Individual performance

**Fig. 5:**
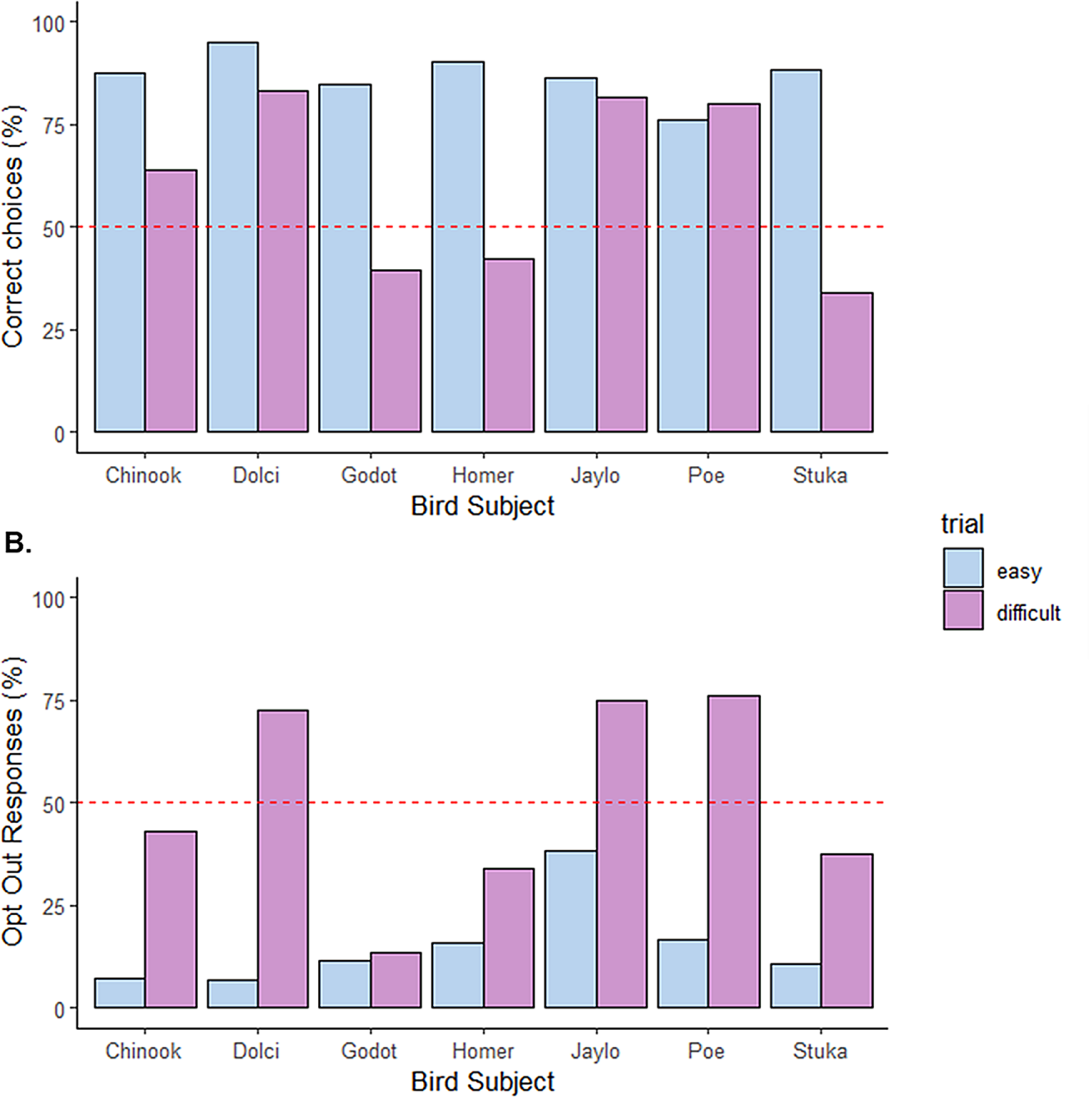
Individual performance. In light blue, individual performance in the easy trials; in blue, individual performance in the difficult trials. Each individual is represented on the x-axis. The dashed red line indicates chance level. **(A)** Percentage of correct responses. **(B)** Percentage of opt-out responses

Based on the analysis of data for each individual (Fig. 5), all seven subjects demonstrated performance that significantly differed from chance during the easy trials, suggesting that the task was easier to complete. For the easy trials, all birds performed at a level significantly above chance: Chinook (87.5%, *p* <.001, *t* = 7.08), Dolci (95.1%, *p* <.001, *t* = 8.58), Godot (84.8%, *p* <.001, *t* = 6.50), Homer (90.3%, *p* <.001, *t* = 10.65), Jaylo (86.2%, *p* <.001, *t* = 5.95), Poe (76.1%, *p* <.001, *t* = 4.10), and Stuka (88.4%, *p* <.001, *t* = 9.89). The highest accuracy was achieved by Dolci, Homer, and Stuka, all exceeding 88% accuracy. These results confirm that the birds were able to perform the easy task well above chance levels.

In contrast, the results from the difficult trials were more mixed. During these trials, three individuals—Dolci, Jaylo, and Poe—performed significantly better than chance. Dolci achieved 83.3% accuracy, which was statistically significant (*p* =.019, t = 2.38), while Jaylo performed at 81.8% (*p* =.033, *t* = 2.18), and Poe achieved 80% accuracy (*p* =.018, *t* = 2.51). These birds showed significantly higher accuracy than would be expected by chance. However, the remaining birds did not perform significantly better than chance on the difficult trials. Chinook showed 64% accuracy, which was above chance but not statistically significant (*p* =.115, *t* = 1.60). Godot’s performance was 39.5%, which was below chance and not statistically significant (*p* =.928, *t* = 0.09). Homer had 42.1% accuracy, which was below chance and also not statistically significant (*p* =.872, *t* = 0.16), and Stuka performed at 34.1%, which was not significantly different from chance (*p* =.989, *t* = 0.02).

Regarding the opt-out behaviour, all individuals exhibited low opt-out rates during the easy trials, with no significant deviation from chance. The opt-out rates were as follows: Chinook (6.98%, *p* = 1, *t* = 0), Dolci (6.8%, *p* = 1, *t* = 0), Godot (11.5%, *p* = 1, *t* = 0), Homer (15.9%, *p* = 1, *t* = 0), Jaylo (38.3%, *p* =.991, *t* = 0.01), Poe (16.4%, *p* = 1, *t* = 0), and Stuka (10.7%, *p* = 1, *t* = 0). No significant evidence was found to suggest that any individual opted out more than expected by chance during the easy trials.

In the difficult trials, the opt-out rates were higher, with three individuals showing significantly greater tendencies to opt out compared to chance. Dolci (72.7%, *p* =.002, *t* = 4.35), Jaylo (75%, *p* <.001, *t* = 6.21), and Poe (70.6%, *p* <.001, *t* = 3.03) all exhibited significantly higher opt-out rates than would be expected by chance. In contrast, Chinook (43.18%, *p* =.855, *t* = 0.18), Godot (13.6%, *p* = 1, *t* = 0), Homer (33.9%, *p* =.995, *t* = 0.01), and Stuka (37.3%, *p* =.986, *t* = 0.03) did not show any significant deviation from chance in their opt-out behaviour during the difficult trials.

**Fig. 6:**
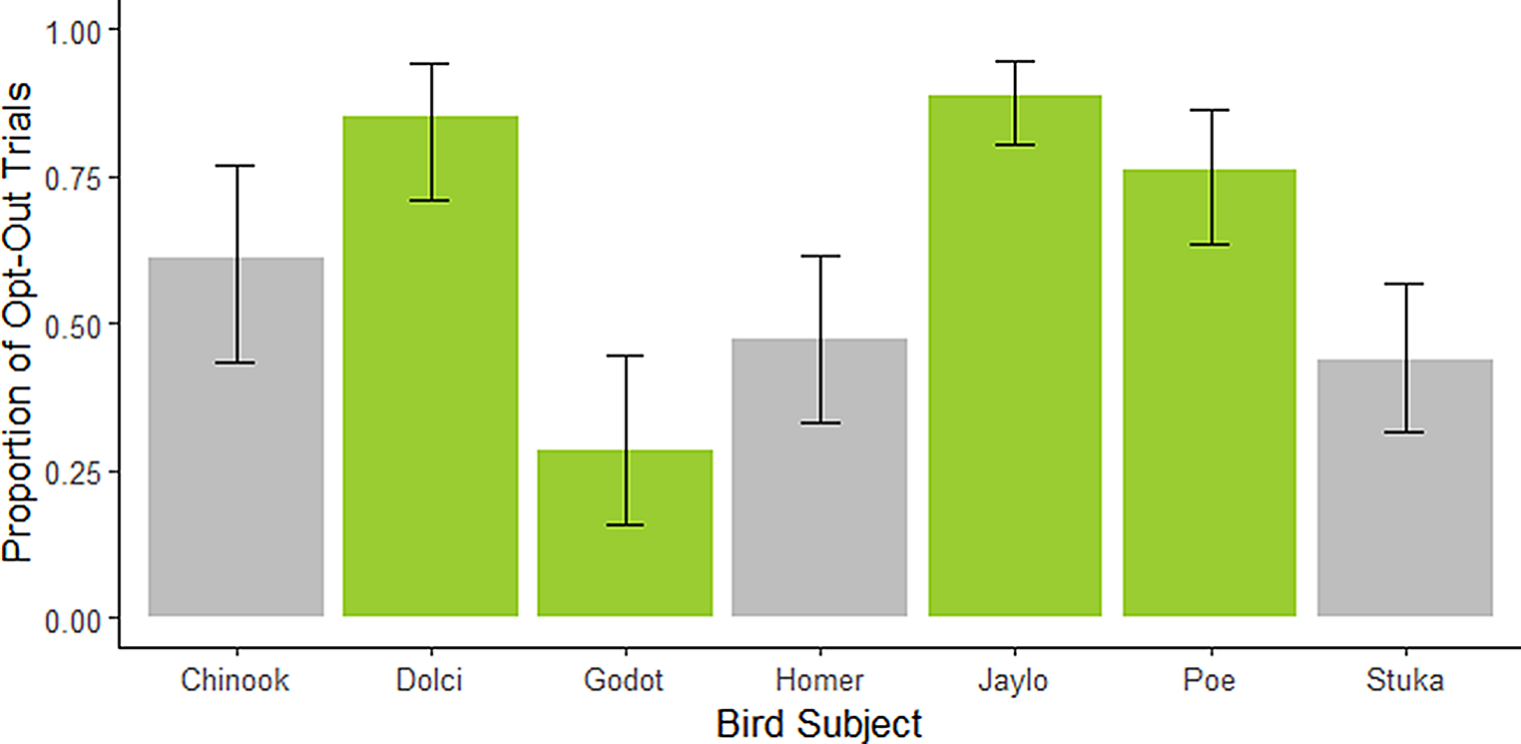
Proportion of Opt-Out Trials by Subject. The bars represent 95% confidence intervals. Subjects that showed a proportion of trials different from chance levels are highlighted in light green. Specifically, Dolci, Jaylo, and Poe exhibited a significant tendency to opt out more than to choose incorrectly. Godot displayed a preference for engaging with the task

In the analysis of opt-out versus incorrect choices, the results revealed varying preferences across subjects (Fig. 6). Dolci, Jaylo, and Poe exhibited a significant tendency to opt out more than to choose incorrectly. Specifically, Dolci opted out in 89.7% of trials (estimate = 0.9, 95% CI: 0.76–0.97, *p* <.001), Jaylo opted out in 87.3% of trials (estimate = 0.87, 95% CI: 0.78–0.94, *p* <.001), and Poe opted out in 76.3% of trials (estimate = 0.76, 95% CI: 0.63–0.86, *p* <.001). These findings suggest that these subjects were more likely to avoid the task rather than making an incorrect choice. In contrast, Godot displayed a preference for engaging with the task, as indicated by choosing incorrectly more often than opting out (estimate = 0.29, 95% CI: 0.16–0.45, *p* <.001), highlighting that Godot was more likely to participate in the task despite the challenge, rather than opting out. Chinook, Homer, and Stuka did not show significant differences between opting out and choosing incorrectly, with p-values greater than 0.05, suggesting these subjects exhibited a more balanced approach to task engagement and opting out. These results illustrate a range of decision-making strategies, from avoidance behaviours to engagement with the task despite challenges.

## Discussion

In our study, we investigated uncertainty monitoring in Eurasian jays, *Garrulus glandarius*. Birds were tested in a working memory food-searching task where they had to locate a highly palatable reward hidden under one of two cups. Cups were shuffled either once (easy treatment), or multiple times (difficult treatment). Additionally, a third, cup– different in colour and size– offered a less preferred food as a safe alternative to engaging with the task. The jays were pre-trained on the task structure before testing. They first learned that red cups contained a preferred reward and the blue cup a less preferred one, and that only one cup could be chosen per trial. They were also familiarized with the red cups being shuffled, introducing uncertainty before the test phase. The only new element during testing was the blue cup as an opt-out option, allowing us to assess metacognitive decision-making—specifically, whether birds would forgo a potentially better reward when uncertain, in favour of a guaranteed but less preferred one.

On average, birds performed with better accuracy in the easy trials than in the difficult ones, confirming the increased complexity of the latter. Moreover, three out of seven birds were more likely to opt out in the difficult trials compared to chance level, suggesting that they recognised the higher risk of error, and consequently preferred the safer, less desirable option. This behaviour parallels findings in monkeys (Beran et al. 2016; Hampton 2001; Sayers et al. 2015), although evidence in bird species remains less consistent. Previous studies indicate that while some birds (large-billed crows, pigeons, and bantams) succeeded in retrospective meta-memory tasks, they failed in prospective tests, where they needed to predict performance before deciding to engage or opt out (Goto and Watanabe 2012; Nakamura et al. 2011). Our results bridge this gap, demonstrating that Eurasian jays can evaluate risks and prospects alongside subjective knowledge, guiding their behaviour based on these assessments. Such cognitive abilities likely benefit natural behaviours such as caching, where accurately assessing memory strength of memory for cache locations is crucial for efficient resource management (Goto and Watanabe 2012). This interpretation in supported numerous studies linking caching behaviours to refined cognitive abilities in corvids (for a review see (Grodzinski and Clayton 2010).

At the individual level, only three birds significantly opted out more often in the difficult trials. Interestingly, these same birds also displayed high accuracy when they chose to engage, suggesting that their decisions were based on a reliable evaluation of their knowledge. This challenges the possibility that opting out was based on simpler associative mechanisms (Inman and Shettleworth 1999; Smith et al. 2003; Sole et al. 2003) —such as the number or duration of shuffles— or by an overall preference for the second-preferred reward, which may have been perceived as more valuable due to its certainty. If such mechanisms were at play, we would have expected consistent opting out and performance at chance level. Instead, these birds showed a higher number of opt-out responses in the difficult trials, and when engaging they also selected the correct option above chance level, indicating engagement stemmed from confidence-based decisions. Moreover, the hypothesis that opting out was driven by a lack of motivation for the primary food reward is not supported by the birds’ food preferences: two of the three individuals that systematically opted out in difficult trials (Dolci and Jaylo) showed an exclusive preference for waxworms over cheese, while the third (Poe) also exhibited a moderate preference for waxworms. This makes it unlikely that opting out was due to reduced incentive value of the preferred food, further reinforcing the interpretation that their decisions reflected an assessment of task difficulty rather than low motivation or simple associative cues.

While this provides strong evidence of metacognitive abilities related to uncertainty monitoring, at least in some individuals, it remains unclear why others did not exhibit the same pattern. We find it unlikely that this disparity results from an actual inability to monitor internal states, as such an ability and its evolutionary advantages would not be exclusive to only some individuals. We propose that individual differences in cognitive and socio-ecological factors might explain this variability. For instance, it must be considered that our participants also received food outside the experimental situation, as part of their regular daily diet or through other experiments. This may have influenced their risk-benefit evaluation, motivating some birds to pursue the task for a better reward despite the risks involved.

Other relevant factors include impulsivity and self-control, which are closely related to metacognition. Studies on Eurasian jays (Miller et al. 2023; Schnell et al. 2022) have shown significant variability in the maximum time that a bird can exert self-control to wait for higher gratification. The variability in opting out may reflect differences in both metacognitive ability and self-control, particularly in how the birds handle uncertainty in the trials. Birds with lower self-control may opt out more frequently in difficult or unpredictable trials, not because they lack the ability to wait for a reward, but because they struggle with the delay involved in uncertain outcomes. This is something to consider in light of individual differences in cognitive and socio-ecological factors that might influence decision-making. It is possible that for some jays, the time interval between trials was too long. This was further exacerbated by the fact that trials followed a pseudo-random order where the difficulty of the next trial was unpredictable, which might have led them to accept the risk of failure while still aiming for the desired reward without additional delays.

Lastly, individual differences in behaviour, often referred to in the literature as ‘personality traits’, likely play an important role (Dingemanse et al. 2002; van Oers et al. 2005). Individual differences on the boldness/shyness continuum have been shown to correlate with risk propensity/aversion in various ecological domains (Bell 2005; Ciacci et al. 2023; Smith and Blumstein 2008) and social contexts (Zoratto et al. 2022). Shy or conservative birds might prioritise the safer, albeit lower quality, option. Conversely, bold birds might be more inclined to take risks, anticipating a potentially better reward. Interestingly, previous experiments on uncertainty monitoring in pigeons and bantams (Nakamura et al. 2011) also reported that only a subgroup of the original sample managed to solve the task. Future studies should focus on assessing the role of individual differences and external factors that could influence bird motivation when evaluating risks and benefits. This would deepen our understanding of metacognition and self-reflection in non-human species and help identify factors mediating behaviour. Understanding the different motivational and socio-environmental factors that could enable individuals to express behaviours associated with uncertainty monitoring could lead to potentially re-evaluate past instances where animals failed to perform successfully on metamemory tasks. This new insight might in fact reveal that previously reported failures were not due to a lack of the underlying ability, but rather to contextual or motivational shortcomings at the time of testing. Such a reassessment could prompt a rethinking of the evolutionary origins of uncertainty monitoring, and consequently self-awareness and metacognition in different non-human species.

## Electronic supplementary material

Below is the link to the electronic supplementary material.

Supplementary Material 1

Supplementary Material 2

Supplementary Material 3

## Data Availability

The data raw data generated during the study are provided in electronic supplementary material.

## References

[CR1] Bell AM (2005) Behavioural differences between individuals and two populations of stickleback (Gasterosteus aculeatus). J Evol Biol 18(2):464–473. 10.1111/j.1420-9101.2004.00817.x 15715852 10.1111/j.1420-9101.2004.00817.x

[CR3] Beran MJ, Perdue BM, Smith JD (2014) What are my chances? Closing the gap in uncertainty monitoring between rhesus monkeys (Macaca mulatta) and capuchin monkeys (Cebus apella). J Experimental Psychology: Anim Learn Cognition 40:303–316. 10.1037/xan0000020 10.1037/xan0000020 PMC4215522 25368870

[CR2] Beran MJ, Perdue BM, Church BA, Smith JD (2016) Capuchin monkeys (Cebus apella) modulate their use of an uncertainty response depending on risk. J Experimental Psychol Anim Learn Cognition 42(1):32–43. 10.1037/xan0000080 10.1037/xan0000080 PMC4710549 26551351

[CR4] Cheke LG, Clayton NS (2012) Eurasian Jays (Garrulus glandarius) overcome their current desires to anticipate two distinct future needs and plan for them appropriately. Biol Lett 8(2):171–175. 10.1098/rsbl.2011.0909 22048890 10.1098/rsbl.2011.0909 PMC3297405

[CR5] Ciacci F, Mayerhoff S, De Petrillo F, Gastaldi S, Brosnan SF, Addessi E (2023) State-dependent risky choices in primates: variation in energy budget does not affect tufted capuchin monkeys’ (Sapajus spp.) risky choices. Am J Primatol 85(10):e23542. 10.1002/ajp.23542 37545247 10.1002/ajp.23542

[CR6] Clayton NS, Dickinson A (1998) Episodic-like memory during cache recovery by scrub Jays. Nature 395(6699) Article 6699. 10.1038/26216 10.1038/26216 9751053

[CR7] Clayton NS, Dickinson A (1999) Scrub Jays (Aphelocoma coerulescens) remember the relative time of caching as well as the location and content of their caches. J Comp Psychol 113(4):403–416. 10.1037/0735-7036.113.4.403 10608564 10.1037/0735-7036.113.4.403

[CR8] Dally JM, Emery NJ, Clayton NS (2006) Food-caching Western scrub-jays keep track of who was watching when. Sci (New York N Y) 312(5780):1662–1665. 10.1126/science.1126539 10.1126/science.1126539 16709747

[CR9] Dingemanse NJ, Both C, Drent PJ, van Oers K, van Noordwijk AJ (2002) Repeatability and heritability of exploratory behaviour in great Tits from the wild. Anim Behav 64(6):929–938. 10.1006/anbe.2002.2006

[CR10] Emery NJ, Clayton NS (2001) Effects of experience and social context on prospective caching strategies by scrub Jays. Nature 414(6862):443–446. 10.1038/35106560 11719804 10.1038/35106560

[CR11] Foote AL, Crystal JD (2007) Metacognition in the rat. Curr Biol 17(6):551–555. 10.1016/j.cub.2007.01.061 17346969 10.1016/j.cub.2007.01.061 PMC1861845

[CR12] Garcia-Pelegrin E, Schnell AK, Wilkins C, Clayton NS (2021) Exploring the perceptual inabilities of Eurasian Jays (Garrulus glandarius) using magic effects. Proc Natl Acad Sci 118(24):e2026106118. 10.1073/pnas.2026106118 34074798 10.1073/pnas.2026106118 PMC8214664

[CR13] Goto K, Watanabe S (2012) Large-billed crows (Corvus macrorhynchos) have retrospective but not prospective metamemory. Anim Cogn 15(1):27–35. 10.1007/s10071-011-0428-z 21681477 10.1007/s10071-011-0428-z

[CR14] Grodzinski U, Clayton NS (2010) Problems faced by food-caching Corvids and the evolution of cognitive solutions. Philosophical Trans Royal Soc B: Biol Sci 365(1542):977–987. 10.1098/rstb.2009.0210 10.1098/rstb.2009.0210 PMC2830244 20156820

[CR15] Grodzinski U, Watanabe A, Clayton NS (2012) Peep to pilfer: what scrub-jays like to watch when observing others. Anim Behav 83(5):1253–1260. 10.1016/j.anbehav.2012.02.018

[CR16] Hampton RR (2001) Rhesus monkeys know when they remember. *Proceedings of the National Academy of Sciences*, *98*(9), 5359–5362. 10.1073/pnas.071600998 10.1073/pnas.071600998 PMC33214 11274360

[CR17] Hartig F, Lohse L (2022) *DHARMa: Residual Diagnostics for Hierarchical (Multi-Level / Mixed) Regression Models* (0.4.6) [Computer software]. https://cran.r-project.org/web/packages/DHARMa/index.html

[CR18] Inman A, Shettleworth SJ (1999) Detecting metamemory in nonverbal subjects: A test with pigeons. J Exp Psychol Anim Behav Process 25(3):389–395. 10.1037/0097-7403.25.3.389

[CR19] Legg EW, Clayton NS (2014) Eurasian Jays (Garrulus glandarius) conceal caches from onlookers. Anim Cogn 17(5):1223–1226. 10.1007/s10071-014-0743-2 24638877 10.1007/s10071-014-0743-2 PMC4138428

[CR20] Miller R, Davies JR, Schiestl M, Garcia-Pelegrin E, Gray RD, Taylor AH, Clayton NS (2023) Social influences on delayed gratification in new Caledonian crows and Eurasian Jays. PLoS ONE 18(12):e0289197. 10.1371/journal.pone.0289197 38055711 10.1371/journal.pone.0289197 PMC10699590

[CR21] Nakamura N, Watanabe S, Betsuyaku T, Fujita K (2011) Do birds (pigeons and bantams) know how confident they are of their perceptual decisions? Anim Cogn 14(1):83–93. 10.1007/s10071-010-0345-6 20665063 10.1007/s10071-010-0345-6

[CR22] Nelson TO (1992) Metacognition: core readings. Allyn and Bacon

[CR23] Ostojić L, Shaw RC, Cheke LG, Clayton NS (2013) Evidence suggesting that desire-state attribution may govern food sharing in Eurasian jays. *Proceedings of the National Academy of Sciences*, *110*(10), 4123–4128. 10.1073/pnas.1209926110 10.1073/pnas.1209926110 PMC3593841 23382187

[CR24] Raby CR, Alexis DM, Dickinson A, Clayton NS (2007) Planning for the future by Western scrub-jays. Nature 445(7130):919–921. 10.1038/nature05575 17314979 10.1038/nature05575

[CR25] Roberts WA, Feeney MC, McMillan N, MacPherson K, Musolino E, Petter M (2009) Do pigeons (Columba livia) study for a test? J Exp Psychol Anim Behav Process 35(2):129–142. 10.1037/a0013722 19364222 10.1037/a0013722

[CR26] Sayers K, Evans TA, Menzel E, Smith JD, Beran MJ (2015) The misbehaviour of a metacognitive monkey. Behaviour 152(6):727–756. 10.1163/1568539X-00003251 26900166 10.1163/1568539X-00003251 PMC4758523

[CR28] Schnell AK, Loconsole M, Garcia-Pelegrin E, Wilkins C, Clayton NS (2021) Jays are sensitive to cognitive illusions. Royal Soc Open Sci 8(8):202358. 10.1098/rsos.202358 10.1098/rsos.202358 PMC8371373 34457330

[CR27] Schnell AK, Boeckle M, Clayton NS (2022) Waiting for a better possibility: delay of gratification in Corvids and its relationship to other cognitive capacities. Philosophical Trans Royal Soc B: Biol Sci 377(1866):20210348. 10.1098/rstb.2021.0348 10.1098/rstb.2021.0348 PMC9620750 36314150

[CR29] Shaw RC, Clayton NS (2012) Eurasian Jays, Garrulus glandarius, flexibly switch caching and pilfering tactics in response to social context. Anim Behav 84(5):1191–1200. 10.1016/j.anbehav.2012.08.023

[CR30] Shaw RC, Clayton NS (2013) Careful cachers and prying pilferers: Eurasian jays (Garrulus glandarius) limit auditory information available to competitors. *Proceedings of the Royal Society B: Biological Sciences*, *280*(1752), 20122238. 10.1098/rspb.2012.2238 10.1098/rspb.2012.2238 PMC3574300 23222444

[CR31] Smith BR, Blumstein DT (2008) Fitness consequences of personality: A meta-analysis. Behav Ecol 19(2):448–455. 10.1093/beheco/arm144

[CR32] Smith JD, Schull J, Strote J, McGee K, Egnor R, Erb L (1995) The uncertain response in the bottlenosed Dolphin (Tursiops truncatus). J Exp Psychol Gen 124:391–408. 10.1037/0096-3445.124.4.391 8530911 10.1037//0096-3445.124.4.391

[CR33] Smith JD, Shields WE, Washburn DA (2003) The comparative psychology of uncertainty monitoring and metacognition. Behav Brain Sci 26(3):317–339. 10.1017/S0140525X03000086 14968691 10.1017/s0140525x03000086

[CR34] Sole LM, Shettleworth SJ, Bennett PJ (2003) Uncertainty in pigeons. Psychon Bull Rev 10(3):738–745. 10.3758/BF03196540 14620372 10.3758/bf03196540

[CR35] Suda-King C (2008) Do orangutans (Pongo pygmaeus) know when they do not remember? Anim Cogn 11(1):21–42. 10.1007/s10071-007-0082-7 17437141 10.1007/s10071-007-0082-7

[CR36] Suda-King C, Bania AE, Stromberg EE, Subiaul F (2013) Gorillas’ use of the escape response in object choice memory tests. Anim Cogn 16(1):65–84. 10.1007/s10071-012-0551-5 22923213 10.1007/s10071-012-0551-5

[CR37] van Oers K, Klunder M, Drent PJ (2005) Context dependence of personalities: Risk-taking behavior in a social and a nonsocial situation. Behav Ecol 16(4):716–723. 10.1093/beheco/ari045

[CR38] Washburn DA, Smith JD, Shields WE (2006) Rhesus monkeys (macaca mulatta) immediately generalize the uncertain response. J Exp Psychol Anim Behav Process 32:185–189. 10.1037/0097-7403.32.2.185 16634662 10.1037/0097-7403.32.2.185

[CR39] Watanabe A, Clayton NS (2016) Hint-seeking behaviour of Western scrub-jays in a metacognition task. Anim Cogn 19(1):53–64. 10.1007/s10071-015-0912-y 26267805 10.1007/s10071-015-0912-y

[CR40] Watanabe A, Grodzinski U, Clayton NS (2014) Western scrub-jays allocate longer observation time to more valuable information. Anim Cogn 17(4):859–867. 10.1007/s10071-013-0719-7 24322875 10.1007/s10071-013-0719-7

[CR41] Yuki S, Okanoya K (2017) Rats show adaptive choice in a metacognitive task with high uncertainty. J Experimental Psychol Anim Learn Cognition 43(1):109–118. 10.1037/xan0000130 10.1037/xan0000130 28045298

[CR42] Zoratto F, Oddi G, Pillitteri S, Festucci F, Puzzo C, Curcio G, Laviola G, Paglieri F, Adriani W, Addessi E (2022) The presence of a potential competitor modulates risk preferences in rats. Behav Process 196:104602. 10.1016/j.beproc.2022.104602 10.1016/j.beproc.2022.104602 35124157

