# Supplementary material for: Uncertainty monitoring in Eurasian jays (Garrulus glandarius)
Source: Anim Cogn. 2025 May 15;28(1):37. doi: 10.1007/s10071-025-01960-3 (PMC12081489; doi:10.1007/s10071-025-01960-3)
Supplement: Supplementary file 2 — Supplementary Material 2 [file 10071_2025_1960_MOESM2_ESM.pdf]

Birds' preferences for each food pairing

Food Choice Summary per Bird (Peanut vs Cheese)

Bird	Total Trials	Peanut	Cheese	% Chose Peanut	% Chose Cheese	Chi-Square p
Chinook	10	9	1	90.0	10.0	0.01140
Dolcinea	8	0	8	0.0	100.0	0.00468
Godot	8	2	6	25.0	75.0	0.15700
Homer	10	10	0	100.0	0.0	0.00157
Jaylo	7	2	5	28.6	71.4	0.25700
Poe	9	2	7	22.2	77.8	0.09560
Stuka	10	10	0	100.0	0.0	0.00157

Food Choice Summary per Bird (Waxworms vs Peanut)

Bird	Total Trials	Waxworms	Peanut	% Chose Waxworms	% Chose Peanut	Chi-Square p
Chinook	13	13	0	100.0	0.0	0.00031
Dolcinea	13	13	0	100.0	0.0	0.00031
Godot	13	13	0	100.0	0.0	0.00031
Homer	23	1	22	4.3	95.7	0.00001
Jaylo	14	14	0	100.0	0.0	0.00018
Poe	15	15	0	100.0	0.0	0.00011
Stuka	12	1	11	8.3	91.7	0.00389

Food Choice Summary per Bird (Waxworms vs Cheese)

Bird	Total Trials	Waxworms	Cheese	% Chose Food 1	% Chose Food 2	Chi-Square p
Chinook	13	13	0	100.0	0.0	0.00031
Dolcinea	15	14	1	93.3	6.7	0.00079
Godot	15	15	0	100.0	0.0	0.00011
Homer	3	3	0	100.0	0.0	0.08330
Jaylo	15	15	0	100.0	0.0	0.00011
Poe	12	8	4	66.7	33.3	0.24800
Stuka	14	14	0	100.0	0.0	0.00018
