# Supplementary material for: Uncertainty monitoring in Eurasian jays (Garrulus glandarius)
Source: Anim Cogn. 2025 May 15;28(1):37. doi: 10.1007/s10071-025-01960-3 (PMC12081489; doi:10.1007/s10071-025-01960-3)
Supplement: Supplementary file 3 — Supplementary Material 3 [file 10071_2025_1960_MOESM3_ESM.pdf]

Table: Trials Included in Individual Performance Analyses (Correct Responses and Opt-Outs)

Bird	Treatment Level	Correct Trials	Opt-Out Trials
Chinook	Easy	40	43
Chinook	Difficult	25	44
Dolci	Easy	41	44
Dolci	Difficult	12	44
Godot	Easy	46	52
Godot	Difficult	38	44
Homer	Easy	62	69
Homer	Difficult	38	56
Jaylo	Easy	58	94
Jaylo	Difficult	11	44
Poe	Easy	46	55
Poe	Difficult	15	51
Stuka	Easy	69	75
Stuka	Difficult	44	67
